# Malaria vectors and transmission dynamics in Goulmoun, a rural city in south-western Chad

**DOI:** 10.1186/1471-2334-9-71

**Published:** 2009-05-23

**Authors:** Clément Kerah-Hinzoumbé, Mallaye Péka, Christophe Antonio-Nkondjio, Issa Donan-Gouni, Parfait Awono-Ambene, Albert Samè-Ekobo, Frédéric Simard

**Affiliations:** 1 Programme National de Lutte contre le Paludisme, N'Djaména, Tchad; 2 Organisation de Coordination pour la lutte contre les Endémies en Afrique Centrale (OCEAC), Yaoundé, Cameroun; 3 Université de Yaoundé 1, Yaoundé, Cameroun; 4 Division de l'Hygiène du Milieu et de l'Assainissement, N'Djaména, Tchad; 5 Faculté de Médecine et de Sciences Pharmaceutiques, Douala, Cameroun; 6 Institut de Recherche pour le Développement (IRD), UR016, Bobo-Dioulasso, Burkina Faso

## Abstract

**Background:**

Knowledge of some baseline entomological data such as Entomological Inoculation Rates (EIR) is crucially needed to assess the epidemiological impact of malaria control activities directed either against parasites or vectors. In Chad, most published surveys date back to the 1960's. In this study, anopheline species composition and their relation to malaria transmission were investigated in a dry Sudanian savannas area of Chad.

**Methods:**

A 12-month longitudinal survey was conducted in the irrigated rice-fields area of Goulmoun in south western Chad. Human landing catches were performed each month from July 2006 to June 2007 in three compounds (indoors and outdoors) and pyrethrum spray collections were conducted in July, August and October 2006 in 10 randomly selected rooms. Mosquitoes belonging to the *Anopheles gambiae *complex and to the *An. funestus *group were identified by molecular diagnostic tools. *Plasmodium falciparum *infection and blood meal sources were detected by ELISA.

**Results:**

Nine anopheline species were collected by the two sampling methods. The most aggressive species were *An. arabiensis *(51 bites/human/night), *An. pharoensis *(12.5 b/h/n), *An. funestus *(1.5 b/h/n) and *An. ziemanni *(1.3 b/h/n). The circumsporozoite protein rate was 1.4% for *An. arabiensis*, 1.4% for *An. funestus*, 0.8% for *An. pharoensis *and 0.5% for *An. ziemanni*. Malaria transmission is seasonal, lasting from April to December. However, more than 80% of the total EIR was concentrated in the period from August to October. The overall annual EIR was estimated at 311 bites of infected anophelines/human/year, contributed mostly by *An. arabiensis *(84.5%) and *An. pharoensis *(12.2%). *Anopheles funestus *and *An. ziemanni *played a minor role. Parasite inoculation occurred mostly after 22:00 hours but around 20% of bites of infected anophelines were distributed earlier in the evening.

**Conclusion:**

The present study revealed the implication of *An. pharoensis *in malaria transmission in the irrigated rice fields of Goulmoun, complementing the major role played by *An. arabiensis*. The transmission period did not depend upon irrigation. Correct use of insecticide treated nets in this area may be effective for vector control although additional protective measures are needed to prevent pre-bedtime exposure to the bites of infected anophelines.

## Background

Despite decades of control efforts, malaria continues to be a major public health concern throughout the world and especially in Africa where 90% of the global cases are recorded [1]. The situation is even worsening with the spread of drug resistant parasites strains, increase of insecticide resistance in vector populations and deleterious economic status of exposed populations. In Chad, malaria affects more than 95% of the overall population (10,044,576 inhabitants) of which more than 15% live in areas where the disease occurs in its epidemic form. Malaria is by far the leading cause of morbidity and mortality recorded in the country, responsible for approximately 25% of total cases at outpatient services, 24.3% of hospitalization and 21.1% of all deaths reported in the hospitals [2]. Estimates of infant mortality in the country show that at least 15% of children under five years of age die each year of malaria (NMCP, unpublished reports). To reduce the disease burden, the National Malaria Control Programme (NMCP) is promoting an integrated approach including mass education, early diagnosis with prompt access to effective treatment and large scale use of insecticide treated nets (ITNs), the latter being the main vector control strategy currently implemented at the community level. Results from several studies showed that high population coverage with ITNs significantly reduces man-vector contact and more importantly, also lead to a decrease in morbidity, mortality and severe malaria among children [3-5]. Nevertheless, successful implementation of this strategy requires prior knowledge of the vector system composition, behaviour and efficiency in malaria transmission. In Chad, little is known about malaria vectors and their relative contribution to the disease transmission. Most entomological surveys have been carried out back to the 1960's [6-8]. These data have never been updated, mostly because of the lack of skilled malaria entomologists working in the country. Moreover, most of these studies focused on anophelines species distribution with no indication on their bionomics or on their involvement in malaria transmission. Yet, this basic information is crucially needed to properly devise and implement malaria vector control interventions and to assess their effectiveness. In an effort to fill this gap, a longitudinal entomological survey was conducted in the irrigated rice-fields area of Goulmoun (south western Chad) to incriminate vector species and to document malaria transmission dynamics. These results will serve as baseline data for the NMCP.

## Methods

### Study area

**Figure 1:**
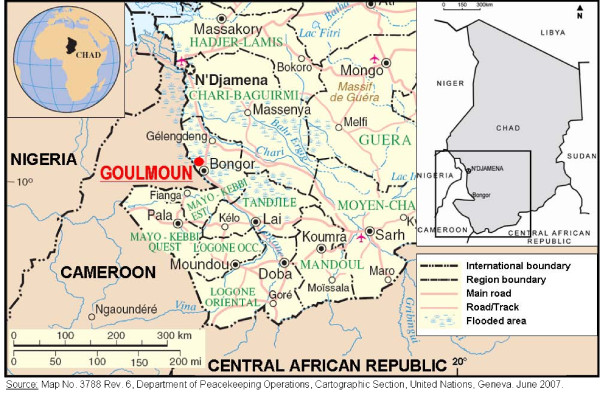
**Schematic map of Chad showing the study area, South West of the country**.

The study was carried out in Goulmoun (10.39°N; 15.20°E), a village situated 5 km from Bongor, the main city of the health district and 250 km south of N'Djamena, the capital city of Chad (Figure 1). The village lies along the banks of river Logone and is surrounded by 800 ha of irrigated rice-fields with two crops per year. During the study period (July 2006 to June 2007), the first cultivation period extended from July to October and the second from March to June. The study area belongs to the Sudanian climatic domain. The rainy season lasts from April to October with mean annual rainfall around 800 mm. Most houses are of traditional types, with mud walls and thatched roof. Family compounds consist of a group of houses separated from each other by surrounding farmlands of sorghum, maize and vegetables. Many inhabitants are also breeders, resulting in year-round occurrence of cows, sheeps and goats in the village. Animal sheds are often situated within the courtyard and at night, small ruminants are usually kept inside the dwellings. During the hot dry season, from March to May, inhabitants usually sleep outdoors, and rarely use mosquito nets. During the rainy season however, when mosquito densities are high, mosquito nets and fumigating coils are frequently used for personal protection. Households ownership of mosquito nets is around 36%, according to the latest demographic and health survey [9]. Resistance to pyrethroid insecticides has recently been reported in the malaria vector, *An. arabiensis *from the area [10]. Likewise, resistance of the parasite, *Plasmodium falciparum *to the antimalarial drugs chloroquine, sulfadoxine-pyrimethamine and amodiaquine was documented [11].

### Mosquito collections and field processing

Adult mosquitoes were collected from July 2006 to June 2007 using two sampling methods: (1) Human Landing Catches (HLC) performed monthly for two consecutive nights, from 18:00 to 6:00 hours, indoors and outdoors in three randomly selected compounds, the same compounds being used throughout the study, and (2) indoor Pyrethrum Spray Collections (PSC) in ten bedrooms randomly selected on each collection date (July, August and October 2006) and different from those used for HLC. Upon collection, anophelines were sorted from other mosquitoes and identified to species according to the morphological identification keys [12,13]. Blood meal spots were collected onto a filter paper after dissecting the midgut of freshly fed resting anophelines. All mosquitoes were kept separately in labelled tubes containing silica gel and frozen at -20°C for laboratory examination.

### Laboratory processing of mosquitoes

All anophelines collected through HLC were tested for the presence of the circumsporozoïte protein (CSP) of *Plasmodium falciparum *using ELISA [14,15]. This *Plasmodium *species was the only one recorded in the study area [11]. The origin of the blood meals collected from freshly fed females was identified by ELISA [16] using human, cow, sheep, chicken, horse, pig and dog antibodies. Species and molecular form identification within the *An. gambiae *complex were carried out by PCR-RFLP [17] and mosquitoes from the *An. funestus *group were molecularly distinguished using diagnostic PCR assays [18].

### Entomological parameters and statistical analysis

The following entomological parameters were determined: (1) the human biting rate (HBR), calculated as the number of mosquitoes biting a person during a given time period (night, month or year); (2) the rate of endophagy, defined as the proportion of mosquitoes caught indoors by HLC over the total number of mosquitoes collected by HLC (indoors and outdoors); (3) the human blood index (HBI) was the proportion of human blood among the total blood meals determined; mixed blood meals being treated as separate blood meals; (4) the CSP rate was the proportion of mosquitoes found with *P. falciparum *CSP over the number of mosquitoes tested and (5) the Entomological Inoculation Rate (EIR), expressed as the number of bites of infected anophelines per person per unit of time and calculated as the product of the HBR by the CSP rate. The overall EIR for a given period was the sum of the EIR contributed by each vector species. Chi-square tests were used to compare different proportions. All tests were performed at the 5% significance level.

### Ethical considerations

The Health Ministry of Chad approved the current research protocol and provided ethical clearance for the implementation of the study (N° 528/PR/PM/MSP/SG/DGAS/DSPLM/DMTNT/PNLAP/05 issued 02/06/05). Likewise, house owners and mosquito collectors gave their informed consent to participate to the study after explanation of the objectives and collections methods through individual discussions and group meetings.

## Results

### Anopheline fauna composition

Overall, nine anophelines species were morphologically identified among the 11,688 specimens collected from July 2006 to June 2007. As shown in Table 1, *Anopheles gambiae *s.l., *An. pharoensis *and *An. funestus *were the most abundant species. *Anopheles ziemanni *was collected biting humans, especially outdoors but was very scarce in PSC, whereas *An. rufipes*, sporadically caught biting humans, was more abundant in PSC.*Anopheles coustani, An. nili, An. squamosus *and *An. wellcomei *were collected only by HLC. There was significant heterogeneity in species diversity and abundance between the three compounds where HLC was implemented (Chi-square = 75.5, ddl = 2, P < 0.001) although the overall biting density was fairly similar across compounds (Table 2). PCR identification of members of species complexes revealed that all of the 2,500 *An. gambiae *s.l tested (2,000 specimens from HLC and 500 from PSC were randomly selected for PCR analysis) consisted only of *An. arabiensis *and all specimens of the *An. funestus *group (218 specimens from HLC and 200 out of 286 specimens collected by PSC) were *An. funestus *s.s.

**Table 1:** Number of anophelines collected in Goulmoun from July 2006 to June 2007.

	Sampling method			
		
Species	Human Landing Catches^a^		Pyrethrum Spray	Total
			
	Indoors	Outdoors	Collection^b^	
*An. gambiae *sl	4,262	3,101	1,611	8,974
*An. pharoensis*	849	947	48	1,844
*An. funestus*	156	62	286	504
*An. ziemanni*	71	120	1	192
*An. rufipes*	6	4	136	146
*An. wellcomei*	8	7	0	15
*An. squamosus*	2	4	0	6
*An. nili*	4	1	0	5
*An. coustani*	0	2	0	2
**Total**	**5,358**	**4,248**	**2,082**	**11,688**

^a ^Total over 144 person-nights;

^b ^Total over 30 room-nights.

**Table 2:** Spatial heterogeneity in mosquito species diversity and abundance collected by Human Landing Catches (HLC) in Goulmoun (July 2006 to June 2007).

	Compound					
	
Species	N°1		N°2		N°3	
			
	Indoors	Outdoors	Indoors	Outdoors	Indoors	Outdoors
*An. gambiae s.l*.	1,133	1,180	1,336	882	1,793	1,039
*An. pharoensis*	233	278	294	389	322	280
*An. funestus*	37	31	46	17	73	14
*An. ziemanni*	29	65	17	29	25	26
*An. rufipes*	1	1	1	0	4	3
*An. wellcomei*	2	3	1	0	5	4
*An. squamosus*	2	3	0	0	0	1
*An. nili*	0	0	3	0	1	1
*An. coustani*	0	1	0	0	0	1
**Total**	**1,437**	**1,562**	**1,698**	**1,317**	**2,223**	**1,369**
	**2,999**		**3,015**		**3,592**	

### Seasonal abundance and biting rates

**Figure 2:**
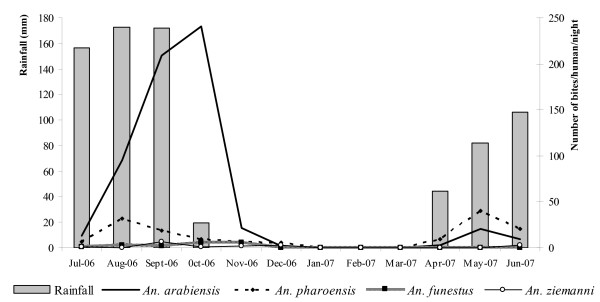
**Monthly rainfall (monthly means averaged over the period 1985 – 2004 obtained from the National Weather Agency) and HBR of the main anopheline species in Goulmoun (July 2006 – June 2007**.

Anophelines were caught throughout the year, with marked variations in abundance according to the season (Figure 2). The average Human Biting Rate (HBR) for the whole study period was 66.7 bites per human per night (b/h/n), consisting of 51 b/h/n for *An. arabiensis*, 12.5 b/h/n for *An. pharoensis*, 1.5 b/h/n for *An. funestus*, 1.3 b/h/n for *An. ziemanni*, the remaining (0.4 b/h/n) being due to the other species. *Anopheles arabiensis *predominated in the collections from July to November while *An*.*pharoensis *was the most abundant species from December to June.

The highest densities for *An. arabiensis *were recorded in October (241 b/h/n) and May (20.6 b/h/n) and the highest densities of *An. pharoensis *in August (31.9 b/h/n) and May (39.8 b/h/n). The second peak observed simultaneously for both species occurred two months after the onset of rainfalls and the transplantation of rice in the irrigated area. *Anopheles ziemanni *and *An. funestus *were observed in the collections from June to December. The highest density for *An. ziemanni *(6.3 b/h/n) was recorded in September. Peak density for *An. funestus *(5.8 b/h/n) occurred in November.

### Host seeking behaviour and feeding preferences

**Figure 3:**
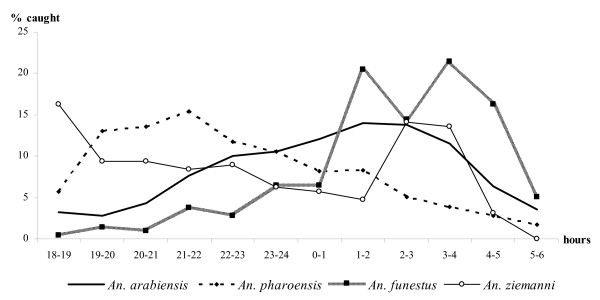
**Night biting cycles of the most abundant anopheline species biting humans in Goulmon (July 2006 – June 2007)**.

The night biting cycles of the four prevalent anopheline species are shown in Figure 3. *Anopheles arabiensis *and *An. funestus *displayed similar biting activities, with more than 60% of their respective bites occurring between 01:00 and 04:00 hours. *Anopheles pharoensis *was more active before midnight, with 70% of the specimens being collected within this period. *Anopheles ziemanni *showed a bimodal distribution, being more aggressive at dusk (43% collected from 18:00 to 22:00 hours) and between 02:00 and 04:00 hours (28% of the collection).

Comparisons between indoor and outdoor catches showed that *An. funestus *was the most endophagic species followed by *An. arabiensis *with 72% (156/218) and 58% (4,262/7,363) of specimens caught indoors, respectively. Throughout the study period, the HBR of both species remained higher inside than outside human dwellings, except in May 2007 when *An. arabiensis *was found to be more abundant outdoors (not shown). On the contrary, *An*. *ziemanni *showed exophagic behaviour, with 63% (120/191) of the specimens being collected outdoors. Overall, 53% (947/1,796) of biting *An. pharoensis *were collected outdoors although the species was found to predominantly bite outdoors or indoors depending on the collection compound (Table 2) and month of collection (not shown), suggesting opportunistic feeding behaviour. No male specimens of any anopheline species was collected by HLC.

Blood meals were processed for 144 *An. arabiensis*, 53 *An. funestus *and 7 *An*. *pharoensis *(Table 3). The Human Blood Index (HBI) was 90.6% (95% CI = 79.3–96.9) for *An. funestus*, 71.4% (95% CI = 29.0–96.3) for *An*. *pharoensis *and 63.9% (95% CI = 56.1–71.7) for *An. arabiensis*. Mixed blood meals included a combination of human and cow bloods (N = 1 in *An. arabiensis*), human+sheep (N = 8 in *An. arabiensis*, N = 1 in *An. funestus*, N = 1 in *An. pharoensis*) and cow+sheep (N = 4 in *An. arabiensis *and N = 1 in *An. funestus*).

**Table 3:** Blood meal sources of indoor resting mosquitoes in Goulmoun (July 2006 – June 2007).

		Mosquitoes fed on each vertebrate host (%)*						
			
Mosquito species	No. of mosquitoes	Human	Bovine	Ovine	Chicken	Equine	Dog	% mixed bloodmeal
*An. arabiensis*	144	63.9	16.7	21.5	0.7	0.7	5.6	9.1
*An. funestus*	53	90.6	3.8	5.7	0.0	1.9	1.9	3.9
*An. pharoensis*	7	71.4	0.0	42.9	0.0	0.0	0.0	14.3

* including mixed blood meals

### *Plasmodium falciparum *infection and entomological inoculation rates

A total of 9,606 mosquitoes collected after landing on humans were processed by ELISA for the detection of *P. falciparum *circumsporozoite protein (Table 4). The CSP rate was similar in *An. arabiensis *and *An. funestus *(1.4% in each species) and somewhat lower in *An. pharoensis *and *An. ziemanni *(0.8 and 0.5%, respectively), although no significant difference was revealed between species (Chi-square with Yates correction = 4.53; ddl = 3; p = 0.21).

**Table 4:** *Plasmodium falciparum *CSP(*) rate and Entomological Inoculation Rate (EIR) in Goulmoun (July 2006 – June 2007).

Mosquito species	No. of mosquitoes	No. CSP positive	% CSP positive (95% CI)	Average Human Biting Rate (No. bites/man/night)	Average number of bites from infected anophelines per man	
					
					per night	per year
*An. arabiensis*	7,363	103	1.4 (1.13–1.67)	51.1	0.72	263
*An. funestus*	218	3	1.4 (0.03–5.45)	1.5	0.02	7.7
*An. pharoensis*	1,796	15	0.8 (0.41–1.26)	12.5	0.10	38.1
*An. ziemanni*	191	1	0.5 (0.00–3.77)	1.3	0.01	2.5

(*) circumsporozoite protein

**Figure 4:**
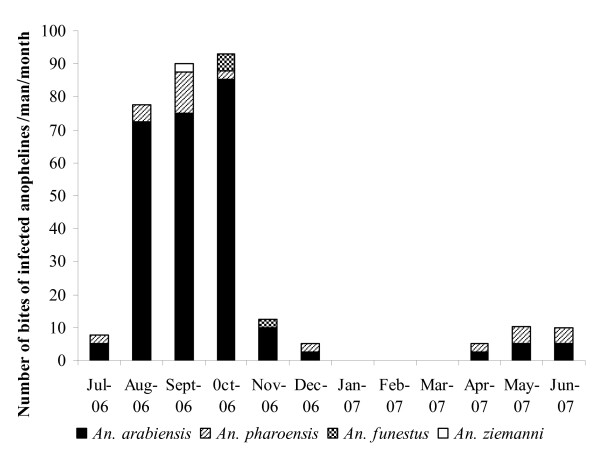
**Monthly Entomological Inoculation Rate for the 4 anopheline species involved in malaria transmission in Goulmoun (July 2006 – June 2007)**.

The overall annual Entomological Inoculation Rate (EIR) for the entire period was 311 bites of infected anophelines/man/year. Malaria transmission occurred from April to December and was due each month to at least two vector species, *An. arabiensis *being always involved (Figure 4). More than 80% of the total EIR was concentrated in the three last months of the rainy season (August to October), with a peak at 93 bites of infected anophelines/man/month in October. *Anopheles arabiensis *was responsible for 84.5% of total malaria transmission, followed by *An. pharoensis *(12.2%), *An. funestus *(2.5%) and *An. ziemanni *(0.8%). From April to June, malaria transmission was more intense outdoors (6.8 bites of infected anophelines/man/month) than indoors (1.7 bites of infected anophelines/man/month). On the contrary, from July to December, the EIR was highest inside the dwellings (55 bites of infected anophelines/man/month) than outside (41 bites of infected anophelines/man/month).

**Figure 5:**
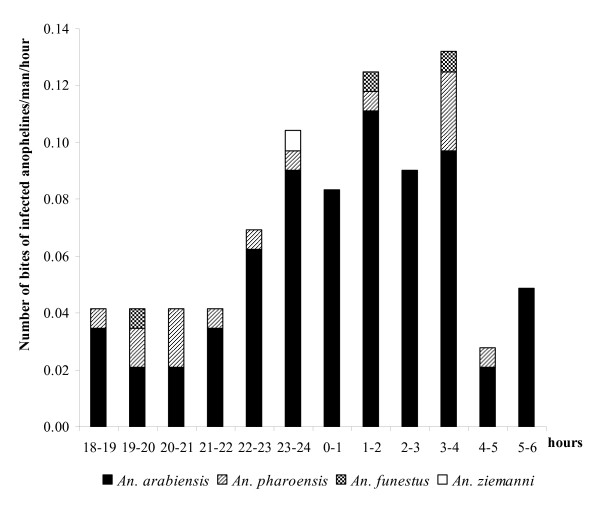
**Hourly distribution of malaria transmission in Goulmoun (July 2006 – June 2007)**.

Analysis of the hourly distribution of EIR showed that 80% of parasite transmission occurred after 22:00 hours, with a peak recorded from 01:00 to 04:00 hours (Figure 5). However, approximately 20% of the bites of infected anophelines were recorded before 22:00 hours, when peoples are still awake and active. Therefore an individual living in Goulmoun throughout the study period may have experienced 66 bites of infected anophelines before going to bed, of which 52 were received during the months of highest transmission (August-October). *Anopheles arabiensis *and *An. pharoensis *were the main vectors species responsible for pre-bedtime parasite transmission.

## Discussion

Successful implementation of a vector control program requires accurate knowledge of the bionomics of the species involved in disease transmission. In Chad, the pattern of malaria transmission is still poorly understood. The establishment of the National Malaria Control Program brought to evidence the urgent need for up-to-date information as current vector control programs are being implemented without any basic knowledge on the target vector populations. To fill this gap, a pilot longitudinal entomological study was conducted in Goulmoun, a village in the south-western part of the country, with the aim to incriminate vector populations and generate information on the dynamics of malaria transmission in this area.

Although sampling methods targeting primarily anthropophilic mosquitoes were used, nine anopheline species out of the thirteen currently known from the country were collected [12]. These nine species were already found in the nearby locality of Bongor and adjacent area by Lacan [6]. Their occurrence in the samples from Goulmoun suggests aquatic habitats suitable for their larval development are available in the study area.

*Anopheles arabiensis, An. pharoensis, An. funestus *and *An*. *ziemanni *constituted more than 90% of the anopheline fauna in Goulmoun. The predominance of *An. arabiensis *in this area is consistent with its distribution throughout Africa [19]. It is however interesting to note that although this pattern of occurrence of *An. arabiensis *in the irrigated rice fields is known from East Africa [20,21], this ecological niche is typically colonized by the Mopti/M form of *An. gambiae *in the dry savannas of West Africa, such as in Mali [22,23] and Burkina Faso [24-26]. In this respect, the current study complements earlier findings from neighbouring areas in Northern Cameroon [27-29] highlighting differences in the distribution of members of the *An. gambiae *complex between Central and West Africa dry savannas areas, and shifting the boundaries of the distribution range of the Mopti/M form of *An. gambiae *west of Cameroon [30,31]. In contrast to *An. arabiensis*, *An. funestus *was not abundant in the collections. Its typical breeding sites consist of large and more or less permanent swamps with emergent vegetation [12]. Such habitats are becoming scarce in the dry savannas areas of Africa because of increasing environmental modifications resulting from human activities and climatic changes [32-34], and rice-fields are generally poorly suitable for the development of *An. funestus *[35,22]. On the other hand, *An*.*pharoensis *was the most abundant anopheline species collected, after *An. arabiensis*. Primarily associated to large swamps with vertical or horizontal vegetation, this species is also very common in irrigated rice-fields throughout the savannas areas in sub-Saharan Africa [35-37]. Overall, the population dynamics of both *An. arabiensis *and *An. pharoensis *appear to be closely linked to the distribution of rainfalls in the area (Figure 2). This suggests that, although permanent breeding is possible in the irrigated rice field paddies or along the banks of river Logone during the dry season, these putative breeding sites contributed to a limited extend to the total mosquito vector population production. However, these could represent refugia where these vector populations can develop at a low rate during the harsh dry season, allowing rapid population expansion at the onset of the next rainy season [38]. In-depth investigations on the larval and adult biology of these species during the dry season should provide invaluable information with relevance for the implementation of targeted vector control strategies based on source reduction.

The present study further indicated that four anophelines species were involved in malaria parasites transmission in Goulmoun. *Anopheles arabiensis *was responsible of 84.5% of the EIR recorded during the full year of the study period (311 bites of infected anophelines/man/year), followed by *An. pharoensis *(12.2%), *An. funestus *(2.5%) and *An. ziemanni *(0.8%). The level of malaria transmission recorded in the current study is consistent with observations in tropical regions where the disease transmission is seasonal and correlates with rainfall patterns [39]. Despite the practice of rice cultivation twice a year, almost all the transmission was recorded during the rainy season suggesting that irrigation for rice cultivation in this area has not a marked effect on the duration of the transmission period. Nevertheless, its real impact on the level of disease transmission remains to be investigated considering the conflicting observations on malaria transmission risk in irrigated rice cultivation areas across the continent [40,41].

Except one survey on malaria transmission in the northern oasis of Faya [42], the current study is the first to evaluate the medical importance of anopheline species collected elsewhere in Chad. The predominant role of *An. arabiensis *in Goulmoun is not surprising since this species is a major vector of malaria in the dry savannas of sub-Saharan Africa [19]. It is interesting to note that, despite lower HBI, its CSP rate was similar to that of *An. funestus*. Although the low number of *An. funestus *specimens analysed by ELISA resulted in large standard error on the CSP rate, this similarity may reflect lower longevity in the latter species, as reported in the Senegal River Basin [37]. The annual EIR due to *An. pharoensis *in Goulmoun (38 bites by infected females/man/year) is not common compared to other studies incriminating this species in malaria transmission across the continent [36,43-45,27,37]. Such a level of parasite transmission is usually associated to high prevalence of the disease [46]. Therefore, one main finding of the current study is that *An. pharoensis *could play a substantial role in malaria transmission when local conditions are favourable. These include high longevity and anthropophilic rate, allowing complete development of *P. falciparum *in the mosquito and its transmission to humans. Our results demonstrated that, in Goulmoun, *An. pharoensis *readily feeds on humans. Furthermore, the infection rate recorded in the current work (0.8%) indicates that *P. falciparum *extrinsic development was completed in *An. pharoensis*. Since the level of anthropophily and the longevity of this species vary spatially across the continent, the presence of sibling species [47] and their possible adaptation to various environments including irrigated rice cultivation areas should be considered.

Malaria transmission in Goulmoun started soon after dusk and continued till daybreak. The peak observed in the second half of the night suggests that correct use of ITNs in this area may be effective for vector control. However, additional protective measures that can be easily accessible and adopted by rural communities are required to prevent pre-bedtime exposure to the bites of infected anophelines. These could include the use of indigenous plants known by local populations for their repellent effects on mosquitoes and other biting insects and, if applicable, targeted source reduction [48].

## Conclusion

The present survey which is the first study on malaria transmission dynamics in Chad, complements previous data on anopheline species distribution in the country. *Anopheles arabiensis *was found to be the main malaria vector in Goulmoun, *An. pharoensis *playing a secondary role, overcoming *An. funestus *generally regarded as a main vector throughout its range. This emergence of *An. pharoensis *as a vector of malaria should be taken into account to plan and implement evidence-based control activities in this area where malaria control is already challenged by *Plasmodium falciparum *resistance to antimalarial drugs and by pyrethroids resistance in *An. arabiensis *populations [11,10]. In this prospect, the data generated in the frame of the current work are important for the national malaria control program managers.

## Competing interests

The authors declare that they have no competing interests.

## Authors' contributions

CKH conceived and designed the study, coordinated its implementation in the field, carried out laboratory procedures, analysed and interpreted data, and drafted the manuscript; MP carried out field experiments, analysed and interpreted data; CAN helped in mosquito collections and contributed to the drafting of the manuscript; IDG participated in the design of the study and supervised fields experiments; PAA helped with laboratory processing, analysis and interpretation of data, and contributed to the drafting of the manuscript; ASE participated in the study design, participated in data analysis and interpretation and provided a critical review of the manuscript; FS participated in the study design, supervised field and laboratory procedures, data analysis and interpretation and revised the manuscript. All authors read and approved the final version of the manuscript.

## Pre-publication history

The pre-publication history for this paper can be accessed here:

http://www.biomedcentral.com/1471-2334/9/71/prepub
